# Efficacy of chronic neck pain self-treatment using press needles: a randomized controlled clinical trial

**DOI:** 10.3389/fpain.2024.1301665

**Published:** 2024-03-22

**Authors:** Kaori Horike, Masatoshi Ukezono

**Affiliations:** ^1^ Comprehensive Human Science, University of Tsukuba, Bunkyo, Japan; ^2^ Product Development Department, Sompo Care Inc., Shinagawa, Japan; ^3^ Department of Developmental Disorders, National Institute of Mental Health, National Center of Neurology and Psychiatry, Kodaira, Japan

**Keywords:** acupuncture, chronic pain, neck pain, self-care, structural equation modeling

## Abstract

**Background:**

Chronic neck pain is common among Japanese individuals, but few receive treatment. This randomized controlled trial aimed to evaluate the efficacy of acupuncture using press needles in the self-treatment of chronic neck pain and preliminarily identify the characteristics of patients likely to benefit from this treatment.

**Methods:**

Fifty participants with chronic neck pain were allocated to receive either press needle or placebo treatment for 3 weeks. The visual analogue scale (VAS) and motion-related VAS (M-VAS) scores for neck pain, Neck Disability Index score, and pressure pain threshold were measured at baseline, after the first session, at the end of the last session, and 1 week after the last session. Changes in the outcomes were analyzed using analysis of variance, and the relationships between the variables were evaluated using structural equation modeling.

**Results:**

Intervention results as assessed by VAS score revealed no significant differences in the ANOVA. A between-groups comparison of M-VAS scores at the end of the last session and baseline showed a significant difference (press needle: −21.64 ± 4.47, placebo: −8.09 ± 3.81, *p* = 0.025, *d* = −0.65). Structural equation modeling revealed a significant pain-reducing effect of press needle treatment (*β* = −0.228, *p* = 0.049). Severity directly affected efficacy (*β* = −0.881, *p* < 0.001). Pain duration, baseline VAS and Neck Disability Index scores were variables explaining severity, while age and occupational computer use were factors affecting severity.

**Conclusion:**

Self-treatment with press needles for chronic neck pain did not significantly reduce the VAS score compared to placebo but reduced the motion-related pain as assessed by M-VAS score. A direct association was observed between pain severity and the effectiveness of press needles, and the impact of age and computer were indirectly linked by pain severity.

**Clinical Trial Registration:**

Identifier UMIN-CTR, UMIN000044078.

## 1 Introduction

Chronic neck pain is common worldwide and has the highest number of years of lifetime prevalence after low back pain (1). Chronic neck pain is the most common complaint among Japanese women and the second most common among Japanese men (2). Predisposing factors for neck pain include age, genetics, obesity, and occupational factors, such as computer use (3, 4). Chronic neck pain has been associated with considerable productivity loss (5) and reportedly decreases quality of life (6). However, only 19.7% of Japanese patients with chronic neck pain receive treatment (7). Consequently, chronic neck pain is not adequately treated.

Acupuncture is widely used to treat chronic pain in clinical practice (4, 8, 9) and has an immediate or short-term effect on chronic neck pain (10–14). The needles used for the treatments range from 13.0 mm to 40.0 mm in length. However, acupuncture using even a 2.5-mm-long superficial needle has been reported to be effective in alleviating myofascial pain in the upper trapezius muscle (15). Patch-type press needles are used clinically in Japan (16). Press needles are tiny acupuncture needles with an invasive depth of less than 1 mm that are fixed to surgical tape. Superficial acupuncture has the advantages of reduced stabbing pain and procedure invasiveness (17). Furthermore, the stimulation is controlled, and press needles can be used for self-treatment. Press needles significantly reduce the intensity of chronic neck pain (18). However, the evidence for their efficacy is minimal (19), and it is necessary to clarify the effects of self-treatment because only a small proportion of patients with chronic neck pain adopt this approach.

The first aim of this study was to evaluate the efficacy of self-administered acupuncture using press needles for alleviating chronic neck pain, assessed using the visual analogue scale (VAS). The second aim was to analyze the relationship between multiple variables using structural equation modeling (SEM) to preliminarily identify the characteristics of patients who are likely to benefit from acupuncture. Identifying these characteristics will help improve the efficiency of acupuncture because its effectiveness varies among individuals (20). In this study, the clinical manifestation of chronic neck pain was subjective and objective pain intensity, functional disability status, and pain duration, as well as sociodemographic characteristics that have been identified as risk factors for neck pain: sex (21, 22), age (21, 23), body mass index (BMI) (4, 21, 24), occupation (4, 21, 22), and exercise habits (4, 21).

## 2 Methods

### 2.1 Study protocol

This clinical trial was registered at UMIN-CTR (trial registration number UMIN000044078). This research was reviewed and approved by the institutional review board of the University of Tsukuba (East 2020-88). Informed consent was obtained from all participants.

### 2.2 Study design

This single-blind, randomized, placebo-controlled, parallel-group trial was conducted from May 2021 to December 2021.

### 2.3 Participants

Fifty-eight students and staff with chronic neck pain were recruited from a medical school in Tokyo and a medical institution in Kanagawa Prefecture. The inclusion criteria were age between 18 and 65 years and neck pain duration of at least 3 months. The exclusion criteria were neuropathic or traumatic neck pain, receiving any treatment for chronic neck pain in the past 2 weeks, and analgesic, muscle relaxant, or psychiatric drug use in the past 1 week.

### 2.4 Randomization and blinding

Participants were assigned to two groups: an intervention group treated with press needles (Needle), and a control group was provided placebo treatment (Placebo). The allocation was stratified according to sex and randomly assigned using a block design. Blocks were generated using an Excel function that returns integer random numbers with a block size of 4 for six patterns. A single-blind design was used in which the participants were blinded. In addition, the assessors for pressure pain threshold (PPT) measurement were blinded.

### 2.5 Interventions

Press needles or placebos application was performed once a week for 3 weeks, and the applied press needles or placebos were retained for 7 days. The maximum number of application points at the tender area from the neck to the scapula was four, regardless of whether the application was performed unilaterally or bilaterally. In both groups, the participants applied the treatment under the guidance of an acupuncturist in the first and second sessions and independently in the third session. The acupuncturist had 10 years of experience and instructed the patients about the points where press needles or placebos should be applied. The points were the tender points in the stiff areas. The acupuncturist indicated the acupoints GB21 and SI14 (25) where tenderness tends to appear, and participants understood to feel the tenderness. Participants applied press needles or placebos after identifying the tenderness via palpation.

**Figure 1:**
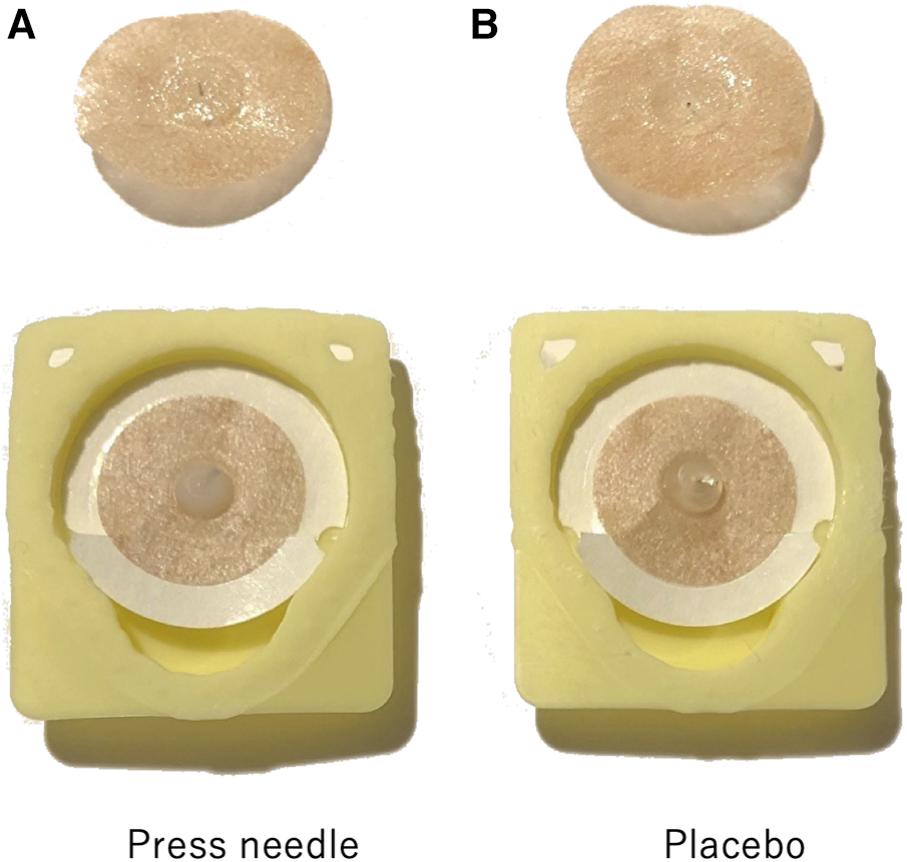
Image of press needle (**A**) and placebo (**B**).

The needles used were Pyonex (SEIRIN Corp., Shizuoka City, Japan), stainless steel needles with a length of 0.6 mm and diameter of 0.15 mm fixed with resin to a 10-mm wide surgical tape (Figure 1A). The placebos had the same packaging as Pyonex, but the needle tips were removed during manufacturing (Figure 1B).

### 2.6 Outcome measures

#### 2.6.1 Primary outcome

The primary outcome was the change in pain intensity on the VAS. The VAS is a 100-mm horizontal line, with 0 (no pain) on the left and 100 (worst pain imaginable) on the right. Participants recorded the subjective intensity of their neck pain on the VAS at baseline (A0), after the first session (A1), at the end of the last session (A2), and 1 week after the last session (A3).

#### 2.6.2 Secondary outcomes

The secondary outcomes were the motion-related VAS (M-VAS) score for neck pain, Japanese version of the Neck Disability Index (NDI-J) score, and PPT of the upper trapezius and levator scapulae, measured at A0, A1, A2, and A3. We also investigated the participants' sex, age, BMI, occupation, exercise frequency, and neck pain duration.

For the M-VAS, participants recorded the subjective intensity of the pain associated with neck motion in six directions (flexion, extension, rotation right/left, lateral flexion right/left) on the VAS.

The NDI-J is commonly used in clinical trials to measure the functional status of patients with neck pain (26, 27). The reliability and validity of the Japanese version have been established (28). Participants completed the questionnaire, comprising 10 items: pain, personal care, lifting, reading, headaches, concentration, work, driving, sleeping, and recreation. Each item was scored on a scale from 0 (no pain) to 5 (worst pain), with a maximum score of 50.

A digital algometer (Wagner Instruments, Greenwich, USA) was used to objectively record the PPT in kg/cm^2^. Measurements were obtained for four trigger points of the upper trapezius and levator scapulae on the left and right sides with the participant seated. The trigger points assessed were equivalent to the GB21 and SI14 acupoints (25, 29). PPT was measured three times at each point, and the average of the second and third measurements was used (30). The compression pressure was increased by 1 kg/cm per second with the rubber tip perpendicular to the skin surface. The participant was asked to say “yes” when pain or discomfort occurred, at which point the compression was immediately stopped.

### 2.7 Sample size calculation

The sample size was calculated using G*Power 3 (RRID: SCR_013726). Based on previously reported effect sizes (10, 12, 18), we obtained the following: *d* = 0.8, 1-*β* = 0.8, and *α* = 0.05. Therefore, the sample size was 57, calculated with a dropout rate of 10%.

### 2.8 Adverse events

Adverse events were investigated at A1 and A2. The acupuncturist informed the participants about adverse events at the beginning of the study. Participants were asked to promptly remove the press needle or a placebo if they experienced itching, rashes, or discomfort at the application point and to report the situation at the subsequent evaluation.

### 2.9 Statistical analyses

Descriptive statistics for each group were calculated for the baseline data. Welch's *t*-test was used to evaluate quantitative variables (age; BMI; VAS, M-VAS, and NDI-J scores; and PPT). Fisher's exact test was used to evaluate categorical variables (sex, occupation, and exercise frequency). Conditions (Needle, placebo) × four endpoints (A0, A1, A2, A3) factorial design ANOVA with repeated measurements was conducted to analyze dependent variables over time. Shaffer's modified sequentially rejective Bonferroni procedure was used for multiple comparisons. Changes in outcomes from A0 were compared between groups using Welch's *t*-test. Individual differences in the effects of acupuncture treatment were analyzed using SEM in an exploratory manner to analyze relationships between variables and identify factors contributing to the change in the VAS score. The model was constructed for the clinical manifestation of chronic neck pain, using the indicators from among the VAS, M-VAS, and NDI-J scores; PPT; and pain duration that were correlated with the change in the VAS score (*r* ≥ 0.2) to define as a latent variable “severity”. Sociodemographic data included sex, age, BMI, exercise frequency, and occupation: sedentary, computer worker (PC), laborer, healthcare worker, driver, and defined as latent variable “characteristics”. In addition, the intervention was used as a variable for modeling. Modeling was performed using the maximum likelihood estimation method, and the model was adjusted by removing unimportant variables if the goodness of fit was not acceptable. Model fit indices of comparative fit index (CIF) ≥0.95, standardized root mean squared residual (SRMR) ≤0.08, and root mean squared error of approximation (RMSEA) ≤0.06 were considered acceptable (31). We also compared the models with the likelihood ration statistics, Akaike information criterion (AIC), Bayesian information criterion (BIC), Goodness-of-fit index (GFI), and Adjusted goodness-of-fit (AGFI). The significance level was set at 5%. All analyses were conducted using R version 4.0.5 (R Core Team. R Foundation for Statistical Computing, Vienna, Austria). The ANOVAKUN package was used for ANOVA and the lavaan package for SEM.

## 3 Results

### 3.1 Participants

**Figure 2:**
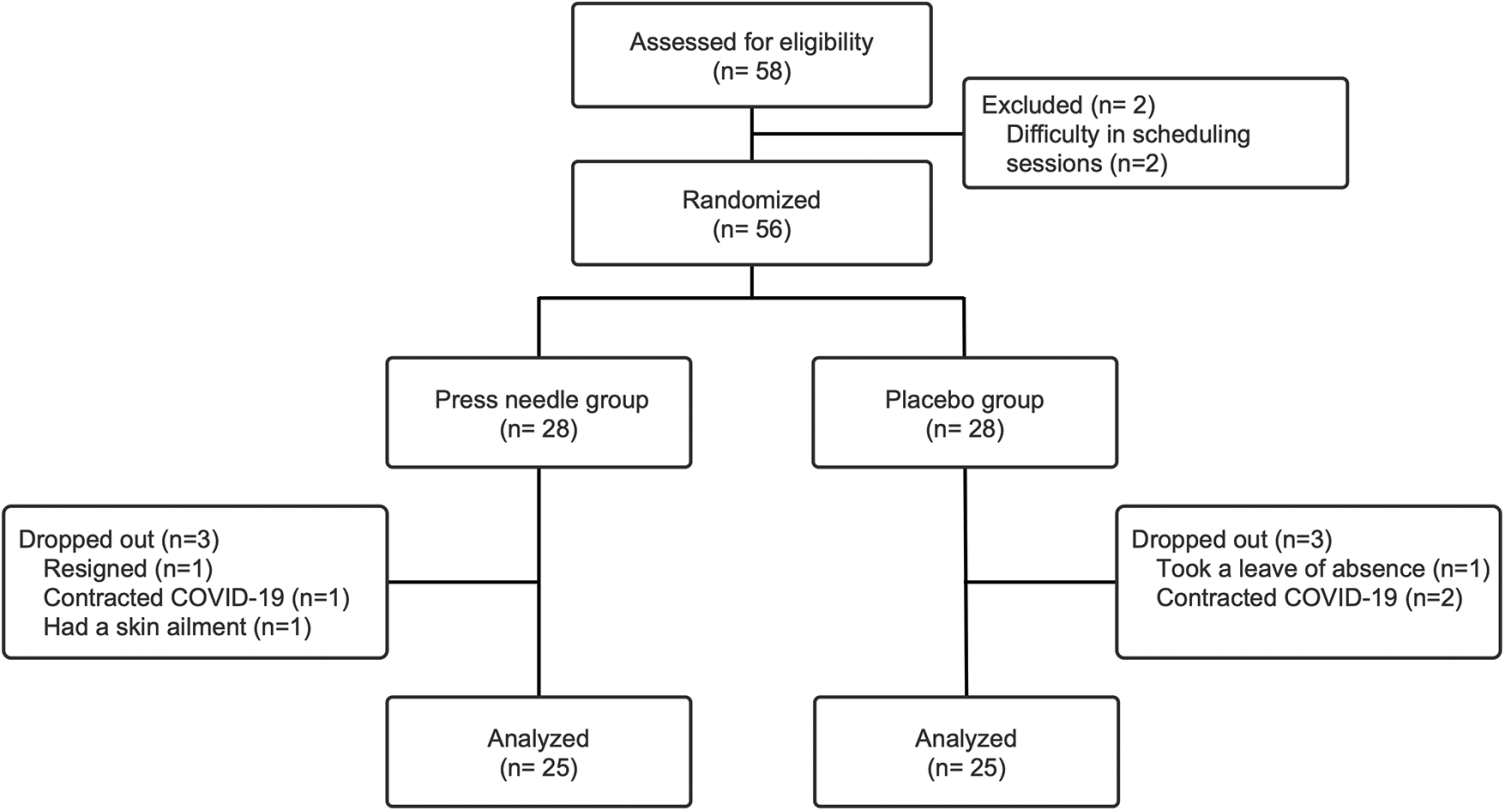
Participant flow chart. COVID-19, coronavirus disease 2019.

Of the 58 eligible participants, two were excluded due to scheduling difficulties; the remaining 56 were assigned to the Needle and Placebo groups. Figure 2 shows the flow of study participants. Three participants dropped out of each group, and 50 participants, 25 in each group, completed the study. The mean age of the participants was 33.1 ± 11.1, and 36 (62%) were women. Table 1 shows the baseline characteristics of the participants, which did not differ significantly between the groups.

**Table 1:** Baseline participant characteristics.

Variables	All participants		Press needle		Placebo		*p*-value
	*n* = 50		*n* = 25		*n* = 25		
Women, *n* (%)	31	(62%)	15	(60%)	16	(64%)	1.000
Age, years	32.96	(±11.28)	34.60	(±12.38)	31.32	(±10.05)	0.309
BMI, kg/m^2^	21.98	(±3.83)	21.76	(±3.52)	22.20	(±4.17)	0.689
Occupation, *n* (%)							
Sedentary	28	(56%)	12	(48%)	16	(64%)	0.393
Computer worker	22	(44%)	11	(44%)	11	(44%)	1.000
Manual laborer	20	(40%)	10	(40%)	10	(40%)	1.000
Healthcare worker	3	(6%)	3	(12%)	0	(0%)	0.235
Driver	4	(8%)	2	(8%)	2	(8%)	1.000
Exercise frequency, *n* (%)							
0 times/week	20	(40%)	9	(36%)	11	(44%)	0.773
1 time/week	13	(26%)	9	(36%)	4	(16%)	0.196
2 or 3 times/week	10	(20%)	2	(8%)	8	(32%)	0.074
4 or 5 times/week	5	(10%)	4	(16%)	1	(4%)	0.349
>6 times/week	2	(4%)	1	(4%)	1	(4%)	1.000
Pain duration, *n* (%)							
3–6 months	6	(12%)	4	(16%)	2	(8%)	0.667
6 months–1 year	6	(12%)	5	(20%)	1	(4%)	0.190
1–5 years	16	(32%)	6	(24%)	10	(40%)	0.364
>5 years	22	(44%)	10	(40%)	12	(48%)	0.776
VAS, mm	51.4	(±23.74)	50.08	(±24.11)	52.72	(±23.78)	0.698
M-VAS, mm	57.55	(±23.27)	59.96	(±22.66)	55.14	(±24.08)	0.470
NDI-J score	7.28	(±4.79)	6.84	(±4.89)	7.72	(±4.75)	0.522
PPT, kg/cm^2^							
Upper trapezius	3.57	(±1.91)	3.49	(±1.55)	3.65	(±2.25)	0.768
Levator scapulae	4.18	(±2.20)	4.24	(±2.11)	4.13	(±2.24)	0.869

Values are means (standard deviations) unless otherwise stated.

BMI, body mass index; M-VAS, motion-related visual analogue scale; NDI-J, Japanese version of the neck disability index; PPT, pressure pain threshold; VAS, visual analogue scale.

### 3.2 Primary outcome

**Figure 3:**
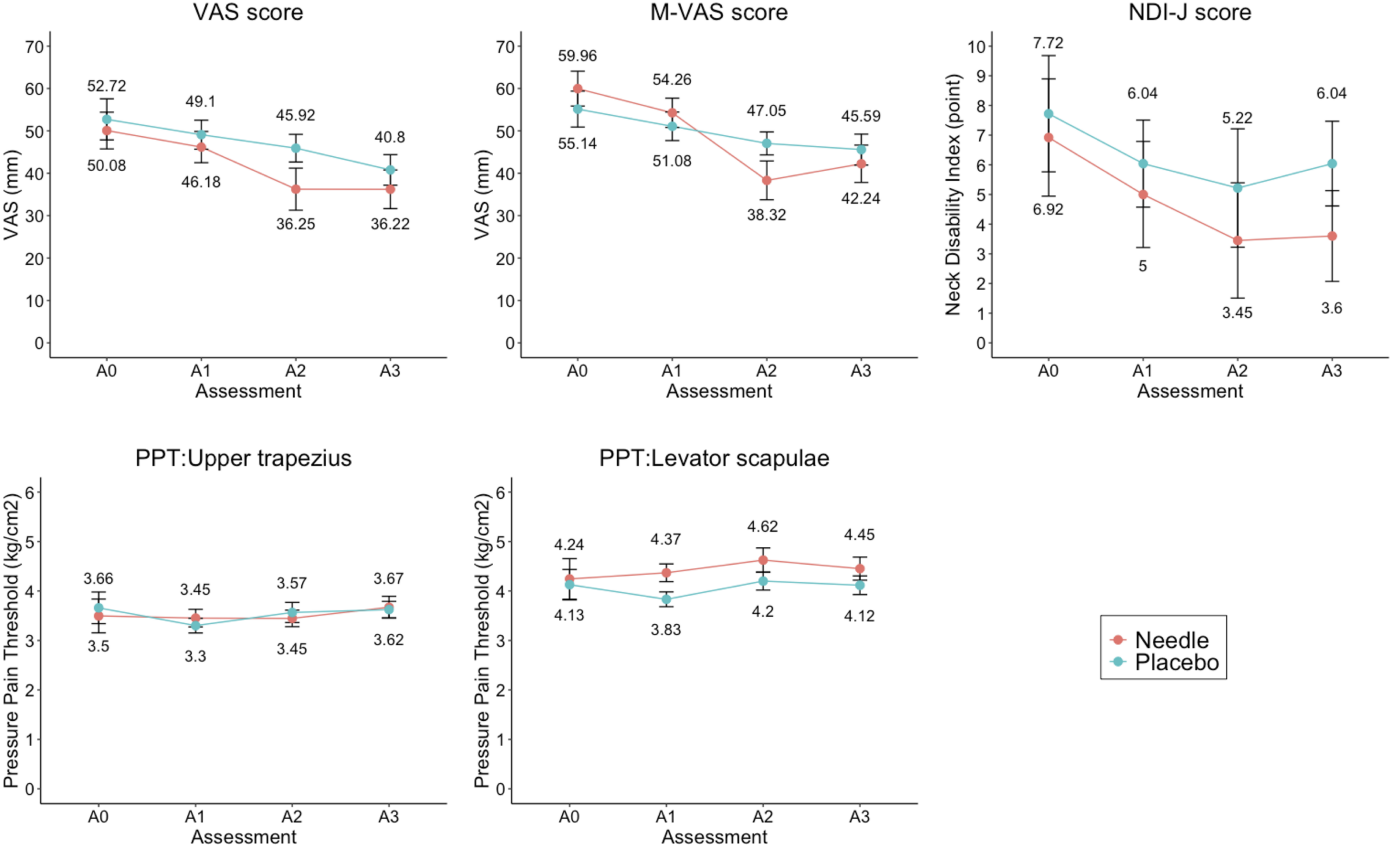
Between-group comparison of outcomes at each endpoint. VAS score changes the most from A0 to A2 (Needle: −13.83 ± 5.10, Placebo: −6.80 ± 4.52), but there is no significant difference between the groups (*p* = 0.307, *d* = 0.292, power = 0.671). M-VAS score significantly differs between A0 and A2 (*p* = 0.025, *d* = 0.653, power = 0.805). There are no significant differences among the other outcomes. A0, baseline; A1, after the first session; A2, at the end of the last session; A3, 1 week after the last session; M-VAS, motion-related visual analogue scale; NDI-J, Japanese version of the neck disability index; Needle, press needle group; Placebo, placebo group; PPT, pressure pain threshold; VAS, visual analogue scale.

Figure 3 shows the results of the two-factor repeated measures ANOVA for the conditions. A significant difference was observed in the endpoint factor [F (3,130) = 8.720, *p* < 0.001, partial *η*^2^ = 0.154] but not in the group factor [F (1, 48) = 0.823, *p* = 0.369, partial *η*^2^ = 0.017] or the interaction effects [F (3,130) = 0.663, *p* = 0.561, partial *η*^2^ = 0.014]. Table 2 shows the comparison of outcomes at A1, A2, and A3 with those at A0 and between-group comparisons. There was no significant between-group difference in the change in the VAS score (*p* = 0.307), while there was a small effect size at A2–A0 (*d* = −0.29).

**Table 2:** Comparison of outcomes at A1, A2, and A3 with those at A0 and between-group comparisons.

Outcome measures			Press needle *n* = 25		Placebo *n* = 25		Mean difference (95% CI)		*t*-value	*df*	*p*-value	Effect size (95% CI)	
			Mean	SE	Mean	SE							
VAS		A1–A0	−3.90	3.08	−3.62	3.98	−0.28	(−10.238, 9.636)	−0.056	45.164	0.956	−0.02	(−0.57, 0.54)
		A2–A0	−13.83	5.10	−6.80	4.52	−7.03	(−20.429, 6.424)	−1.031	47.305	0.307	−0.29	(−0.85, 0.27)
		A3–A0	−13.86	4.28	−11.92	4.21	−1.94	(−13.829, 9.829)	−0.323	47.984	0.748	−0.09	(−0.65, 0.46)
M-VAS		A1–A0	−5.70	2.91	−4.06	3.13	−1.64	(−10.025, 6.824)	−0.384	47.755	0.703	−0.11	(−0.66, 0.45)
		A2–A0	−21.64	4.47	−8.09	3.81	−13.55	(−25.369, −1.735)	−2.307	46.841	0.025	−0.65	(−1.22, −0.08)
		A3–A0	−17.72	4.45	−9.55	4.32	−8.17	(−20.322, 4.119)	−1.317	47.961	0.194	−0.37	(−0.93, 0.19)
NDI-J		A1–A0	−1.92	3.08	−1.68	0.79	−0.24	(−2.263, 1.783)	−0.239	45.237	0.812	−0.07	(−0.62, 0.49)
		A2–A0	−4.16	5.28	−2.92	0.96	−1.24	(−4.109, 1.629)	−0.869	47.560	0.389	−0.25	(−0.8, 0.31)
		A3–A0	−3.32	4.18	−1.68	0.89	−1.64	(−4.093, 0.813)	−1.344	47.825	0.185	−0.38	(−0.94, 0.18)
PPT	Traps	A1–A0	−0.04	0.26	−0.36	0.24	0.32	(−0.293, 1.093)	0.918	47.658	0.363	0.26	(−0.3, 0.81)
		A2–A0	−0.06	0.29	−0.09	0.30	0.03	(−0.813, 0.813)	0.097	47.958	0.924	0.02	(−0.53, 0.58)
		A3–A0	0.18	0.31	−0.02	0.26	0.20	(−0.597, 0.996)	0.496	46.815	0.622	0.14	(−0.41, 0.7)
	LSM	A1–A0	0.13	0.31	−0.29	0.23	0.42	(−0.362, 1.162)	1.102	43.690	0.283	0.31	(−0.25, 0.86)
		A2–A0	0.38	0.36	0.07	0.27	0.31	(−0.588, 1.188)	0.674	44.357	0.493	0.20	(−0.36, 0.75)
		A3–A0	0.22	0.35	−0.02	0.27	0.24	(−0.674, 1.074)	0.523	45.103	0.596	0.15	(−0.4, 0.71)

A0, baseline; A1, after the first session; A2, end of the last session; A3, 1 week after the last session; df, degrees of freedom; LSM, levator scapulae; M-VAS, motion-related visual analogue scale; NDI-J, Japanese version of the neck disability index; PPT, pressure pain threshold; SE, standard error; Traps, upper trapezius; VAS, visual analogue scale; CI, confidence interval.

### 3.3 Secondary outcomes

**Figure 4:**
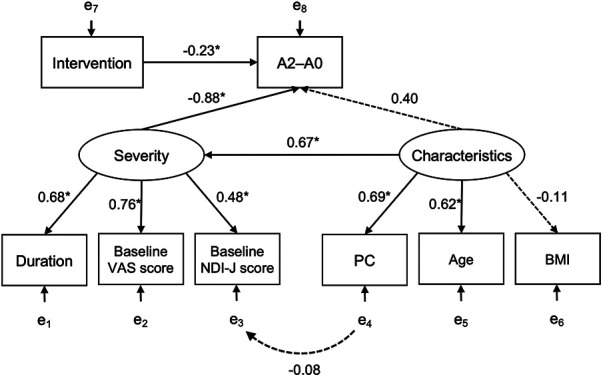
Structural model of the relationship between the change in VAS score from A0 to A2 and each variable. The estimated model fits the sample data (*χ*^2^ = 17.286, *p* = 0.435, comparative fit index = 0.994, root mean squared error of approximation = 0.018, standardized root mean squared residual = 0.079). Intervention and severity are significantly related to A2–A0 (intervention: *β* = −0.228, *p* = 0.049; severity: *β* = −0.881, *p* < 0.001). Characteristics do not directly affect A2–A0 (*p* = 0.227) but indirectly affect it via severity (*β* = 0.673, *p* = 0.031). * *p* < 0.05; A2–A0, change in VAS score from A0 to A2; BMI, body math index; Characteristics, latent variable; Duration, neck pain duration; Intervention, press needle or placebo group; NDI-J, Japanese version of the neck disability index; PC, occupational computer use; Severity, latent variable; VAS, visual analogue scale.

There were no significant differences in secondary variables, such as the M-VAS, NDI-J score, or PPT. However, for the M-VAS, the interaction showed a significant trend [F (3,124) = 2.775, *p* = 0.052, partial *η*^2^ = 0.055]. Multiple comparisons of M-VAS showed that the Needle group was significantly different at A2 and A3 compared to A0 [A2–A0 = −21.64, *t* (24) = 4.842, *p* < 0.001] [A3–A0 = −17.72, *t* (24) = 3.983, *p* = 0.002], while no differences were observed for the Placebo group. Moreover, the change in the M-VAS score in the A2–A0 differed between groups with a medium effect size (*p* = 0.025, *d* = −0.65) (Table 2). NDI-J score also showed a small effect size in A2–A0 (*d* = 0.25) and A3–A0 (*d* = 0.38) comparisons (Table 2). Using SEM, a model assuming A2–A0 from pain severity, characteristics, and intervention was constructed. For severity, the M-VAS and PPT were excluded from the model because they showed a negligible correlation with A2–A0. The model consisted of 3 indicators: VAS, NDI-J, and pain duration. Characteristics consisted of all indicators of sociodemographic characteristics in the first model: sex, age, BMI, exercise, sedentary, PC, laborer, healthcare worker, and driver. The first model showed poor fit to the data (*χ*^2^ = 116.572, *df* = 75, *p* = 0.002, CFI = 0.634, SRMR = 0.155, RMSEA = 0.105, AIC = 2,346.912, BIC = 2,402.361, GFI = 0.751, AGFI = 0.652). The model was adjusted by excluding factors with negligible correlations to A2–A0 and severity (sex, exercise, sedentary, laborer, healthcare worker, and driver). The path diagram of the adjusted model is shown in Figure 4 and the results of the adjusted model are summarized in Table 3. The model fit the sample data (*χ*^2^ = 17.286, *df* = 17, *p* = 0.435, CFI = 0.994, SRMR = 0.079, RMSEA = 0.018, AIC = 2,060.402, BIC = 2,094.819, GFI = 0.919, AGFI = 0.828). Severity was significantly regressed A2–A0 (*β* = −0.881, *p* < 0.001) and loaded significantly on the VAS score (*β* = 0.763, *p* < 0.001), pain duration (*β* = 0.679, *p* < 0.001), and NDI-J score (*β* = 0.482, *p* = 0.012). The characteristics had a non-significant path coefficient to A2–A0 (*p* = 0.227) but a significantly regressed severity (*β* = 0.673, *p* = 0.031). The characteristics loaded significantly on PC (*β* = 0.689, *p* < 0.001) and age (*β* = 0.622, *p* < 0.001), while the association with BMI was not significant (*p* = 0.540). PC correlated with NDI-J item headache, but the path was not significant (*p* = 0.629). Intervention regressed significantly on A2–A0 (*β* = −0.228, *p* = 0.049).

**Table 3:** Results of the covariance structure analysis.

Variables	Estimate	SE	*z*-value	*p*-value
Latent variables				
Severity				
VAS	13.254	3.637	3.645	0.000
Pain duration	0.511	0.146	3.503	0.000
NDI-J	1.670	0.665	2.513	0.012
Characteristics				
Age	6.950	1.920	3.619	0.000
PC	0.342	0.088	3.872	0.000
BMI	−0.399	0.651	−0.612	0.540
Regressions				
Severity∼Characteristics	0.910	0.421	2.163	0.031
NDI-J∼PC	−0.724	1.497	−0.484	0.629
A2–A0∼				
Intervention	−11.078	5.638	−1.965	0.049
Severity	−15.860	4.409	−3.597	0.000
Characteristics	9.738	8.066	1.207	0.227

A2–A0, the change in VAS score; BMI, body mass index; NDI-J, Japanese version of the neck disability index; PC, occupational computer use; SE, standard error; VAS, visual analogue scale.

### 3.4 Adverse events

Adverse events occurred in 28% (Needle: 36%, Placebo: 20%) and 12% (12% each) of participants at A1 and A2, respectively. The reported symptoms were itching (A1: three participants per group, A2: three participants per group), rash (A1: three participants in Needle, none in Placebo; A2: no participants in either group), and discomfort (A1: three participants in Needle, none in Placebo; A2: no participants in either group). All symptoms caused by skin contact with the surgical tape or puncture irritation of the stainless steel needle were minor and disappeared under observation or by removal of the press needle or placebo.

## 4 Discussion

This randomized controlled trial examined the efficacy of acupuncture using press needles in the self-treatment of chronic neck pain and preliminarily identified the characteristics of patients who are likely to benefit from this treatment. The results of the intervention based on VAS scores revealed no significant differences between baseline and the other three endpoints, although a small effect size was observed. However, the M-VAS score showed a significant difference in A2 compared to A0 only in the Needle group in multiple comparisons. Self-treatment with press needles was effective in reducing motion-related neck pain. Pain severity was directly correlated with treatment efficacy, while the factors of age and PC worker showed indirect effects as factors affecting severity.

The mechanism of acupuncture-induced analgesia involves pain perception modulation through central sensitization and antinociception (32, 33). Motion-related neck pain is nociceptive pain caused by the somatosensory response of the musculoskeletal system, including the muscles, joints, and tendons, to mechanical stimulation (34). In contrast, chronic pain as measured by VAS scores, involves brain circuits related to emotion and memory in addition to the sensory system (35). Therefore, we speculate that the motion-related pain which was measured by the degree of M-VAS score could reflect acupuncture-induced pain relief well. In addition, the effect was immediate but not sustained because there was a significant difference between the Needle and Placebo groups at the end of the last session but not 1 week after the last session. Press needles seem to have a briefer effect of treatment than general acupuncture needles, which show effects for durations ranging from 1 week to several months (10–15).

A major aim of treating chronic pain is to reduce disability and improve daily functioning (36, 37). The effects on the M-VAS scores in this study demonstrated a reduction in motion-related pain, leading to an improvement in daily function. Furthermore, the use of press needles for self-management of chronic pain is helpful. Indeed, it has been reported that self-management of chronic pain improves the psychological well-being of patients and is cost-effective (38–41). Self-treatment in the form of strength training, stretching, and walking reportedly improves chronic neck pain among office workers (42–45). Press needle requires minimal effort and is simply applied to the tender area. Therefore, the press needle is a time-efficient tool to enhance self-efficacy with minimal effort (46, 47).

SEM analysis revealed a significant pain reduction effect of the press needles (*β* = −0.228, *p* = 0.049), and a direct association between the pain severity and the effectiveness of press needles (*β* = −0.881, *p* < 0.001). The variables explaining severity observed in this study were the VAS score at A0, neck pain duration, and NDI-J score at A0, all of which were significant, with the VAS score having the greatest impact (*β* = 0.763). In 2019, Witt et al. stated that “patients reporting more severe pain at baseline experiencing more benefit from acupuncture compared to either sham-control or non-acupuncture control” (20). Thus, severity at baseline influenced treatment efficacy, even in self-administered acupuncture with 0.6-mm superficial needles.

Among the variables measured as characteristics, the impact of age and PC were indirectly linked through the pain severity. In previous studies, age (21, 23) and PC worker factors are known risk factors for neck pain (4, 21, 22). We observed no association between BMI and chronic pain severity, although this association has been reported previously (24, 48, 49). This might have been due to the small percentage of obese participants in our study and their low BMI level; the participants' BMI was 21.98 ± 3.83. Knowledge of the factors affecting severity may help clinicians better select patients who will benefit from self-administered acupuncture with press needles.

The limitation of this study is its small sample size. While there were no significant differences in VAS or NDI, the treatment group compared to the placebo had small effect sizes (VAS: A2–A0: *d* = 0.29) (NDI: A2–A0: *d* = 0.25, A3–A0: *d* = 0.38). A trend toward greater improvement in the treatment group suggests that a larger sample is needed. The control group receiving placebo treatment could be another possible drawback. In 2018, Vickers et al. stated that “the effect sizes are close to 0.5 in comparison to no acupuncture control and 0.2 for comparisons with sham” (50). In this study, the placebo was not invasive but provided a pressure stimuli; this may have reduced the effect size compared to press needles, which have an invasiveness of only 0.6 mm. Further limitations could be that the analyzed factors did not encompass all possible clinical manifestations and sociodemographic characteristics of chronic neck pain. Therefore, future studies should adjust for various confounding factors, including psychosocial factors, as, for example, the association between psychological job strain, social support, work environment, and neck pain has been previously noted (21).

This study examined whether superficial press needles are more effective than placebo in the self-treatment of chronic neck pain. This treatment did not improve VAS scores but did reduce motion-related pain. The treatment effect was immediate and not sustained. In addition, a direct association was observed between the pain severity and the effectiveness of press needles. The VAS score at baseline, pain duration, and the NDI-J score directly affected the severity, while age and PC worker factors were indirectly linked through pain severity. Press needle is a helpful self-care tool for managing chronic neck pain.

## Data Availability

The raw data supporting the conclusions of this article will be made available by the authors, without undue reservation.
